# A rare human infection of *Raoultella ornithinolytica* in a diabetic foot lesion

**DOI:** 10.4103/0256-4947.75794

**Published:** 2011

**Authors:** Yalcin Solak, Enes Elvin Gul, Huseyin Atalay, Nejdet Genc, Halil Z. Tonbul

**Affiliations:** a From the Department of Nephrology, Meram School of Medicine, Selcuk University, Konya, Turkey; b Department of Cardiology, Meram School of Medicine, Selcuk University, Konya, Turkey; c Department of Clinical Microbiology and Infectious Diseases, Meram School of Medicine, Selcuk University, Konya, Turkey

## To the Editor:

*Raoultella ornithinolytica* is a gram-negative encapsulated aerobic bacillus belonging to family Enterobacteriaceae that is found in aquatic environments, fish, and insects.^1^ The most distinctive feature of this bacterium is its ability to convert histidine to histamine in scombroid fish. Consequently, infection with this microorganism causes redness and flushing on the skin. Histamine fish poisoning is caused by histamine-producing bacteria (HPB).*R ornithinolytica* is a histamine-producing bacterium that subsequently causes fish poisoning.^2^ Human infections related to R *ornithinolytica* are exceedingly rare. Here, we report R *ornithinolytica* isolated from the wound of diabetic foot in a patient with predialytic chronic kidney disease (CKD).

**Figure 1:**
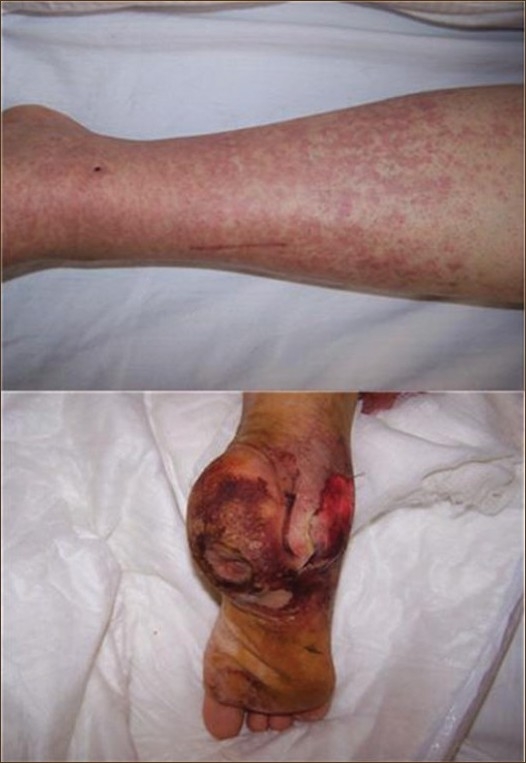
Maculopapular rash in the lower leg (upper) and a disfiguring deep diabetic wound in the lateral malleoli and bottom of the heel (lower).

A 44-year-old woman presented with complaints of weakness, dyspnea on exertion, and foot swelling. She had a previous history of diabetes mellitus, hypertension, CKD, and hypothyroidism. Current medications included insulin aspart, diltiazem, and levothyroxin. On admission, blood pressure was 160 mm Hg. On physical examination, the patient appeared to be in moderate respiratory distress. The findings of a cardiovascular examination were normal. The respiratory rate was 30 breaths per minute and auscultation of lungs revealed bibasilar crackles. There was moderate peripheral edema in the legs and a diabetic foot lesion on the bottom of the right heel. A maculapapular rash was evident in the lower limbs (**Figure 1**). The patient suffered from a fever (38.5°) and non-ketotic hyperglycaemia (glucose, 619 mg/dL). Intensive insulin treatment was initiated. She had uncontrolled diabetes with a 15.2% HbA_1C_ level. Her biochemistry panel and blood counts revealed a white blood cell count of 12.4×10 ^9^/L, hemoglobin of 8.5 g/dL, hematocrit of 27.2%, platelet count of 345×10 ^9^/L, serum sodium of 128 mEq/L, potassium of 5.0 mEq/L, urea of 105 mEq/L, and creatinine of 2.6 mg/dL. Inflammation markers were high—erythrocyte sedimentation rate (ESR) was 32 m/h, C-reactive protein and procalcitonin levels were 32.5 mg/L and 1.01 ng/mL, respectively.

Throat, blood and urine cultures were obtained and empiric antimicrobial treatment with piperacillin and tazobactam was commenced. The blood and urine cultures were negative. Fever persisted under antimicrobial treatment and the culture from the diabetic foot lesion yielded a gram-negative bacillus R *ornithinolytica* (biotype 77563289). The isolated bacterium was positive for lactose, indole, and ornithine and was identified with the help of Vitek 2 System (bioMérieux, Marcy l’Etoile, France). An antibiogram for R *ornithinolytica* demonstrated susceptibility only to ertapenem, levofloxacin, and tigecycline. Antimicrobial therapy was switched to tigecycline 100 mg once daily. With this therapy, she became afebrile and the rashes in the legs disappeared; also, her clinical status significantly improved.

R *ornithinolytica* has been isolated from the gut of the fish, termites, and aquatic environments.^1^ An ability to convert histidine to histamine, leading to fish poisoning had been reported previously.^2^ This bacterium was first described in 1989 by Sakazaki et al.^3^ There are only three case reports of human infection by *R ornithinolytica* in the literature. The first patient was an 82-year-old woman in whom the microorganism caused an enteric fever-like syndrome, and the organism was isolated from blood.^1^ The second patient was a 97-year-old woman who presented with a giant renal cyst, which caused colic obstruction. The fluid culture isolated from the cyst was positive for R *ornithinolytica*.^4^ The third reported case was of R *ornithinolytica* bacteremia in an infant with visceral heterotaxy.^5^ Only this case showed marked skin flushing, which was possibly related to a histamine reaction.

The present case illustrates for the first time, isolation of R *ornithinolytica* from a diabetic foot wound in a patient with many comorbidities and an association with a rash. The distinctive feature of our case was R *ornithinolytica* that was markedly resistant to antimicrobial agents compared with previous reports. R *ornithinolytica* has been shown to be resistant to ampicillin and other commonly used antibiotics.^6^ In the abovementioned cases, skin rash possibly due to a histamine reaction was reported in only one case. We speculate that maculopapular rash in the proximity of diabetic wound may also be related to the histamine-producing characteristic of R *ornithinolytica*. Despite the existence of myriad causes of maculopapular rashes on the legs, the disappearance of the rash after fever ceases with tigecycline treatment may suggest a causal link between the rash and R *ornithinolytica*.

## References

[1] Morais VP, Daporta MT, Bao AF, Campello MG, Andrés GQ (2009). Enteric fever-like syndrome caused by Raoultella ornithinolytica (Klebsiella ornithinolytica). J Clin Microbiol.

[2] Kanki M, Yoda T, Tsukamoto T, Shibata T (2002). Klebsiella pneumoniae produces no histamine: Raoultella planticola and Raoultella ornithinolytica strains are histamine producers. Appl Environ Microbiol.

[3] Kosako Y, Tamura K, Sakazaki R, Miki K (1989). Klebsiella ornithinolytica sp. nov., formerly known as ornithine-positive Klebsiella oxytoca. Curr Microbiol.

[4] Vos B, Laureys M (2009). Giant renal cyst as cause of colic obstruction. Rev Med Brux.

[5] Mau N, Ross LA (2010). Raoultella ornithinolytica bacteremia in an infant with visceral heterotaxy. Pediatr Infect Dis J.

[6] Hostacká A, Klokocníková (2001). Antibiotic susceptibility, serum response and surface properties of Klebsiella species. Microbios.

